# Genetic testing of *FUS*, *HTRA2,* and *TENM4* genes in Chinese patients with essential tremor

**DOI:** 10.1111/cns.13305

**Published:** 2020-03-20

**Authors:** Ya‐Ping Yan, Cong‐Ying Xu, Lu‐Yan Gu, Bo Zhang, Ting Shen, Ting Gao, Jun Tian, Jia‐Li Pu, Xin‐Zhen Yin, Bao‐Rong Zhang, Guo‐Hua Zhao

**Affiliations:** ^1^ Department of Neurology The Second Affiliated Hospital Zhejiang University School of Medicine Hangzhou China; ^2^ Department of Neurology The Second Affiliated Hospital of Jiaxing University Jiaxing China; ^3^ Department of Surgery The Second Affiliated Hospital Zhejiang University School of Medicine Hangzhou China; ^4^ Department of Neurology The Fourth Affiliated Hospital Zhejiang University School of Medicine Hangzhou China

**Keywords:** essential tremor, *FUS*, genetic analysis, *HTRA2*, *TENM4*

## Abstract

**Introduction:**

Essential tremor (ET) is one of the most prevalent movement disorders. The genetic etiology of ET has not been well defined although a significant proportion (≥50%) are familial cases. Linkage analysis and genome‐wide association studies (GWASs) have identified several risk variants. In recent years, whole‐exome sequencing of ET has revealed several specific causal variants in *FUS* (p.Q290X), *HTRA2* (p.G399S), and *TENM4* (c.4324 G>A, c.4100C>A, and c.3412G>A) genes.

**Objective:**

To investigate the genetic contribution of these three genes to ET, the protein‐coding sequences of *FUS, HTRA2*, and *TENM4* were analyzed in a total of 238 ET patients and 272 controls from eastern China using direct Sanger sequencing.

**Results:**

We identified two synonymous coding single nucleotide polymorphisms (SNPs), rs741810 and rs1052352 in *FUS*, and three previously reported synonymous SNPs, rs11237621, rs689369, and rs2277277 in *TENM4*. No nonsynonymous exonic variants were identified in these subjects. We found that the frequency of the rs1052352C allele was significantly higher (*P* = .001) in the ET group than in the control group.

**Conclusion:**

Overall, our findings suggest that rs1052352 of *FUS* might contribute to ET risk in Chinese population.

## 1 INTRODUCTION

Essential tremor (ET) is a common adult‐onset movement disorder with an unknown etiology. The prevalence increases with advancing age, being 0.9% at all ages and 4% in individuals aged ≥ 65 years.^1^ Once considered a benign monosymptomatic motor disease, ET is now known to include many other nonmotor symptoms, with cognitive, psychiatric, dementia, and sensory features,^2^ which are similar to Parkinson's disease (PD). However, unlike PD, there is still no breakthrough in this field except for classical drugs such as propranolol.^3^ Deficiency of disease‐specific biomarkers for ET has resulted in high misdiagnosis rates; usually, it is misdiagnosed as PD.^4^

Despite its high rates of prevalence and frequent cases of misdiagnosis, our understanding of the pathogenesis of ET continues to be limited. Although ≥50% of patients have family history, the genetic etiology of ET is still unclear.^5^ This situation highlights a need for more genetic studies on this complex disorder to improve our understanding with the purpose of seeking better treatments.

Although a strong genetic hereditary component exists,^5^ only a few pathogenic risk loci or genes have been discovered. Genome‐wide scanning of several ET families has identified the first three associated loci (*ETM1, ETM2*, *and ETM3*).^6^, ^7^, ^8^ Later, another five potential ET susceptibility genes (*LINGO1, SLC1A2, PPARGC1A, STK32B*, and *CTNNA3*) were also found by genome‐wide association studies (GWASs).^9^, ^10^, ^11^ Since 2012, pathogenic variants of *FUS, HTRA2*, and *TENM4* have identified to be responsible for familial ET through whole‐exome sequencing.^12^, ^13^, ^14^

Overall, to explore the roles of these three genes in ET, we sequenced all exons of *FUS*, *HTRA2*, and *TENM4* in 238 ET and 272 healthy control subjects from eastern China.

## 2 MATERIALS AND METHODS

A total of 238 unrelated Han Chinese patients were recruited at the Department of Neurology, The Second Affiliated Hospital of Zhejiang University, with a diagnosis of ET based on recommendations of the Consensus Statement of the Movement Disorders Society.^15^ Definite ET required bilateral postural tremor (with or without kinetic tremor) of the hands and forearms, with a duration of longer than 5 years. Exaggerated physiological tremor, psychogenic‐like tremor, the presence of other abnormal neurological signs, and some identified causes of other disease were all excluded. However, there are some changes in the diagnostic criteria in 2018.^16^ Among the 238 cases, 49.58% of patients exhibited a positive family history. Another 272 healthy controls, matched for age and sex (Table 1), were recruited. The study had been conducted in accordance with the Declaration of Helsinki. Written informed consent was obtained from each subject, and the current study was approved by the local ethics committees.

**TABLE 1:** Case‐control dataset included in this study

	ET		Controls	
	FET	SET	Controls 1	Controls 2
Number of subjects	n = 118	n = 120	n = 132	n = 140
Mean age at onset (y) ± SD (range)	44.81 ± 15.17 (6‐71)	49.11 ± 15.33 (16‐79)		
Mean disease duration (y) ± SD (range)	12.35 ± 12.30 (1‐54)	9.54 ± 11.17 (1‐54)		
Gender				
Male	58	63	62	75
Female	60	57	70	65

Genomic DNA was extracted from white cells using standard phenol‐chloroform method. Polymerase chain reaction (PCR) amplification and sequencing of *FUS, HTRA2*, and *TENM4* gene coding region were performed using the ABI 3100 Automated Sequencer for both 238 patients and 272 controls. Primer and amplification conditions are available upon request.

Statistical analysis was performed using SPSS 16.0 (SPSS Inc). Descriptive statistics (range, mean, and standard deviation) for both cases and controls were determined (Table 1). Pearson's chi‐squared test was used to compare the allele frequency between ET cases and controls for each SNP (Table 2). The odds ratio (OR) with 95% confidence interval for each SNP was calculated. *P* < .05 was considered statistical significant for all analysis. And the obtained five *P* values were corrected by false discovery rate (FDR) test. The statistical power was performed by the Genetic Power Calculator software (Gauderman, 2002). All of the above five SNPs were in Hardy‐Weinberg equilibrium.

**TABLE 2:** Allele frequencies for the five SNPs of *FUS* and *TENM4* genes in ET patients and controls

SNP	Alleles	FET, Number of alleles (frequency)	Control_1_, Number of alleles (frequency)	*P*_1_, OR_1_ (95% CI)	SET, Number of alleles (frequency)	Control_2_, Number of alleles (frequency)	*P*_2_, OR_2_ (95% CI)	ET, Number of alleles (frequency)	Control, Number of alleles (frequency)	*P*, OR (95%)
rs741810	Allele C	204 (86.44%)	222 (84.09%)	.460, 1.028 (0.956‐1.106)	187 (77.92%)	231 (82.50%)	.189, 0.944 (0.866‐1.030)	391 (82.14%)	453 (83.27%)	.634, 0.986 (0.932‐1.044)
	Allele A	32 (13.56%)	42 (15.91%)	.460, 0.852 (0.557‐1.304)	53 (22.08%)	49 (17.50%)	.189, 1.262 (0.891‐1.787)	85 (17.86%)	91 (16.73%)	.634, 1.068 (0.816 −1.397)
rs1052352	Allele T	187 (79.23%)	233 (88.26%)	**.006**, 0.898 (0.830‐0.971)	193 (80.42%)	244 (87.14%)	**.037**, 0.923 (0.854‐0.997)	380 (79.83%)	477 (87.68%)	**.001**, .910 (0.862‐0.962)
	Allele C	49 (20.76%)	31 (11.74%)	**.006**, 1. 768 (1. 169‐2.675)	47 (19.58%)	36 (12.86%)	**.037**, 1.523 (1.023‐2.269)	96 (20.17%)	67 (12.32%)	**.001**, 1.638 (1.229‐2.181)
rs11237621	Allele C	104 (44.07%)	132 (50.00%)	.185, 0.881 (0.731‐1.063)	125 (52.08%)	158 (56.43%)	.321, 0.923 (0.787‐1.082)	229 (48.11%)	290 (53.31%)	.097, 0.902 (0.799‐1.020)
	Allele T	132 (55.93%)	132 (50.00%)	.185, 1.119 (0.948‐1.320)	115 (47.92%)	122 (43.57%)	.321, 1.100 (0.912‐1.327)	247 (51.89%)	254 (46.69%)	.097, 1.111 (0.981‐1.259)
rs689369	Allele C	196 (83.05%)	234 (88.64%)	.072, 0.937 (0.872‐1.007)	203 (84.58%)	239 (85.36%)	.805, 0.991 (0.922‐1.066)	399 (83.82%)	473 (86.95%)	.157, 0.964 (0.916‐1.015)
	Allele T	40 (16.95%)	30 (11.36%)	.072, 1.492 (0.961‐2.315)	37 (15.42%)	41 (14.64%)	.805, 1.053 (0.699‐1.586)	77 (16.18%)	71 (13.05%)	.157, 1.239 (0.920‐1.670)
rs2277277	Allele C	195 (82.63%)	230 (87.12%)	.160, 0.948 (0.880‐1.022)	209 (87.08%)	242 (86.43%)	.826, 1.008 (0.942‐1.078)	404 (84.87%)	472 (86.76%)	.387, 0.978 (0.930 ‐1.029)
	Allele T	41 (17.37%)	34 (12.88%)	.160, 1.349 (0.887‐2.05 2)	31 (23.92%)	38 (13.57%)	.826, 0.952 (0.612‐1.481)	72 (15.13%)	72 (13.24%)	.387, 1.143 (0.844 −1.547)

Note

Characters in boldface represent the *P* value reached the significance level (*P* < .05).

## 3 RESULTS

Two synonymous coding SNPs (Table 2, rs741810 and rs1052352) were detected after sequencing of the *FUS* gene. For rs1052352, a significant difference in allelic distribution was found between ET and controls (*P* = .001), both for familial ET vs controls and for sporadic ET vs controls (Table 2). After correction by FDR test, the difference was still significant (*P* = .005) (Table S1). The minor allele (allele C) of rs1052352 seemed to be less common in controls than in patients (12.32% vs 20.17%) (Table 2). And the statistical power of rs1052352 between ET and controls was more than 0.8 (Table 3), while the statistical power between familial/sporadic ET and controls also reached the level of 0.8 (Tables S2 and S3). The minor allele frequencies for rs741810 observed in the present study appeared to be similar to the SNP database (https://www.internationalgenome.org/1000‐genomes‐browsers/).

**TABLE 3:** Power calculation of five loci in ET patients and controls (dominant and recessive models)

SNP	Model	MAF	Control	ET	CON per case	OR	Power
rs741810	Dominant	0.173	272	238	1.143	1.118	0.139
	Recessive					0.855	0.071
rs1052352	Dominant	0.160				1.800	**0.996**
	Recessive					10.651	**0.999**
rs11237621	Dominant	0.491				1.225	0.298
	Recessive					1.382	0.660
rs689369	Dominant	0.145				1.301	0.508
	Recessive					1.733	0.318
rs2277277	Dominant	0.141				1.327	0.563
	Recessive					0.321	0.506

Note

The positive locus identified in this study was marked in bold font.

Abbreviations: CON, controls; ET, essential tremor; MAF, minor allele frequency; OR, odds ratio; SNP, single nucleotide polymorphism.

Our sequencing study of the *HTRA2* gene in 238 patients and 272 controls did not reveal any exonic variant. We did not observe the *HTRA2* S399 mutation and the A141S polymorphism that have been described in previous studies.^17^, ^18^

Three previously reported synonymous SNPs in *TENM4* were also identified in our study, rs11237621, rs689369, and rs2277277. They have been reported in the NCBI database, dbSNP. No novel missense coding variants of *TENM4* were detected in our study. The allele frequency of these SNPs between ET patients and controls did not reach statistical significance in our samples (all *P* > .05) (Table 2).

## 4 DISCUSSION

In this study, *FUS, HTRA2*, and *TENM4* genes were sequenced in 238 ET patients and 272 controls of Chinese origin. Five reported SNPs, rs741810 and rs1052352 in *FUS*, and rs11237621, rs689369, and rs2277277 in *TENM4*, were detected.

The *FUS* gene, once considered as a fusion oncogene, is located on chromosome 16. Apart from its role in various cellular functions*,* it is also proved to be associated with the etiology of certain neurodegenerative diseases.^19^ A nonsense mutation (p.Q290X) in this gene was supposed to cause familial ET in a large Canadian family through exome sequencing. Another two novel variants (p.R216C and p.P431L) were also identified in this study.^12^ In 2013, a novel *FUS* gene variant of p.Met392Ile was detected by Tan et al, which caused increased risk of ET in patients of Chinese ethnicity.^20^ Other researchers detected a heterozygous p.Arg377Trp substitution in an ET family,^21^ while some other studies detected various known SNPs, such as rs741810, rs1052352, and rs138901914.^22^ In our present study, we similarly did not detect any novel *FUS* mutations, except for two synonymous exonic SNPs, rs741810 and rs1052352. Allele frequencies of rs741810 did not differ significantly between cases and controls, which was consistent with previous observations.^22^, ^23^, ^24^, ^25^, ^26^ However, the frequency of rs1052352 was significantly higher in cases than in controls. Our findings suggest that rs1052352 might be a risk factor for ET in eastern China, but this link is not proved by other studies.^23^, ^24^, ^25^, ^26^ Nonetheless, whether this synonymous variant contributes to ET is still unknown. The number of our ET sample is not large enough to reach a solid conclusion.

The mitochondrial high‐temperature–regulated A2 (HtrA2) was found to play essential roles in mitochondrial function and cellular apoptosis regulation.^27^ Gulsuner et al reported a six‐generation family with ET and ET with parkinsonism carrying *HTRA2* p.G399S variant, suggesting its role in both PD and ET.^13^ Additionally, some novel variants, such as c.427C>G, c.106C>T, IVS5+29T>A, and c.G77A were identified in PD patients.^28^, ^29^ However, the study conducted by Tzoulis et al did not identify an association between the p.G399S mutation and ET^30^ and their study also found no exonic variants in the Chinese PD patients.^31^ In our study, no exonic mutation was detected in ET patients. Thus, *HTRA2* mutations are unlikely to be a causative gene for ET or PD in eastern China.

TENM4, mostly distributed in the cerebellum, plays roles in the regulation of axon guidance and central myelination.^32^ In 2015, three distinct pathogenic variants in *TENM4* (c.4100C>A, c.4324 G>A, and c.3412G>A) were identified in three families of Spanish origin.^14^ One year later, Houle et al detected numerous rare missense variants in patients, and no significant association was discovered between ET patients and controls in a Canadian population.^33^ In the current study, we detected three known SNPs, rs11237621, rs689369, and rs2277277 in *TENM4*. The allele frequencies of these SNPs did not differ significantly between ET patients and controls (all *P* > .05). Our study also did not support TENM4 as a causative gene for ET.

## 5 CONCLUSION

In conclusion, our data show that rs1052352 in the *FUS* gene increases the risk for ET in the eastern China and that the other two genes, *HTRA2* and *TENM4*, do not show an association with ET. Our study is the first to reveal that rs1052352 (*FUS*) is associated with ET in eastern China. However, due to the limited number of our sample and racial heterogeneity, more studies are needed to completely elucidate whether *FUS* gene is a risk factor for ET in other populations. Additionally, as the criteria of ET varied, the new diagnostic criteria should be used by more neurologists. Further functional studies are also required to elucidate the potential role played by *FUS* variants in the development of this disease.

## CONFLICT OF INTEREST

The authors declare no conflict of interest.

## Supporting information

Table S1‐S3

## References

[1] Shanker V . Essential tremor: diagnosis and management. BMJ. 2019;366:l4485. 31383632 10.1136/bmj.l4485

[2] Fois AF , Briceno HM , Fung V . Nonmotor symptoms in essential tremor and other tremor disorders. Int Rev Neurobiol. 2017;134:1373‐1396. 28805576 10.1016/bs.irn.2017.05.010

[3] Ferreira JJ , Mestre TA , Lyons KE , et al. MDS evidence‐based review of treatments for essential tremor. Mov Disord. 2019;34:950‐958. 31046186 10.1002/mds.27700

[4] Molparia B , Schrader BN , Cohen E , et al. Combined accelerometer and genetic analysis to differentiate essential tremor from Parkinson's disease. PeerJ. 2018;6:e5308. 30042899 10.7717/peerj.5308 PMC6055592

[5] Jiménez‐Jiménez FJ , Alonso‐Navarro H , García‐Martín E , Lorenzo‐Betancor O , Pastor P , Agúndez JAG . Update on genetics of essential tremor. Acta Neurol Scand. 2013;128:359‐371. 23682623 10.1111/ane.12148

[6] Gulcher JR , Jónsson Þ , Kong A , et al. Mapping of a familial essential tremor gene, FET1, to chromosome 3q13. Nat Genet. 1997;17:84‐87. 9288103 10.1038/ng0997-84

[7] Higgins JJ , Pho LT , Nee LE . A gene (ETM) for essential tremor maps to chromosome 2p22‐p25. Mov Disord. 1997;12:859‐864. 9399207 10.1002/mds.870120605

[8] Shatunov A , Sambuughin N , Jankovic J , et al. Genomewide scans in North American families reveal genetic linkage of essential tremor to a region on chromosome 6p23. Brain. 2006;129:2318‐2331. 16702189 10.1093/brain/awl120

[9] Stefansson H , Steinberg S , Petursson H , et al. Variant in the sequence of the LINGO1 gene confers risk of essential tremor. Nat Genet. 2009;41:277‐279. 19182806 10.1038/ng.299 PMC3740956

[10] Thier S , Lorenz D , Nothnagel M , et al. Polymorphisms in the glial glutamate transporter SLC1A2 are associated with essential tremor. Neurology. 2012;79:243‐248. 22764253 10.1212/WNL.0b013e31825fdeed PMC3398434

[11] Müller SH , Girard SL , Hopfner F , et al. Genome‐wide association study in essential tremor identifies three new loci. Brain. 2016;139:3163‐3169. 27797806 10.1093/brain/aww242 PMC5382938

[12] Merner N , Girard S , Catoire H , et al. Exome sequencing identifies FUS mutations as a cause of essential tremor. Am J Hum Genet. 2012;91:313‐319. 22863194 10.1016/j.ajhg.2012.07.002 PMC3415547

[13] Unal GH , Gulsuner S , Mercan FN , et al. Mitochondrial serine protease HTRA2 p. G399S in a kindred with essential tremor and Parkinson disease. Proc Natl Acad Sci U S A. 2014;111(51):18285‐18290. 25422467 10.1073/pnas.1419581111 PMC4280582

[14] Hor H , Francescatto L , Bartesaghi L , et al. Missense mutations in TENM4, a regulator of axon guidance and central myelination, cause essential tremor. Hum Mol Genet. 2015;24:5677‐5686. 26188006 10.1093/hmg/ddv281 PMC4692992

[15] Deuschl G , Bain P , Brin M . Consensus statement of the Movement Disorder Society on Tremor. Ad Hoc Scientific Committee. Mov Disord. 1998;13(Suppl 3):2‐23. 10.1002/mds.870131303 9827589

[16] Bhatia KP , Bain P , Bajaj N , et al. Consensus statement on the classification of tremors. from the task force on tremor of the International Parkinson and Movement Disorder Society. Mov Disord. 2018;33:75‐87. 29193359 10.1002/mds.27121 PMC6530552

[17] Strauss KM , Martins LM , Plun‐Favreau H , et al. Loss of function mutations in the gene encoding Omi/HtrA2 in Parkinson's disease. Hum Mol Genet. 2005;14:2099‐2111. 15961413 10.1093/hmg/ddi215

[18] Jones JM , Datta P , Srinivasula SM , et al. Loss of Omi mitochondrial protease activity causes the neuromuscular disorder of mnd2 mutant mice. Nature. 2003;425:721‐727. 14534547 10.1038/nature02052

[19] Deng H , Gao K , Jankovic J . The role of FUS gene variants in neurodegenerative diseases. Nat Rev Neurol. 2014;10:337‐348. 24840975 10.1038/nrneurol.2014.78

[20] Wu Y‐R , Foo JN , Tan LCS , et al. Identification of a novel risk variant in the FUS gene in essential tremor. Neurology. 2013;81:541‐544. 23825177 10.1212/WNL.0b013e31829e700c

[21] Wood H . Movement disorders: novel FUS gene variants linked to essential tremor. Nat Rev Neurol. 2013;9:418. 23877643 10.1038/nrneurol.2013.143

[22] Hopfner F , Stevanin G , Muller SH , et al. The impact of rare variants in FUS in essential tremor. Mov Disord. 2015;30:721‐724. 25631824 10.1002/mds.26145

[23] Hedera P , Davis TL , Phibbs FT , Charles PD , LeDoux MS . FUS in familial essential tremor ‐ the search for common causes is still on. Parkinsonism Relat Disord. 2013;19:818‐820. 23660545 10.1016/j.parkreldis.2013.04.009

[24] Labbé C , Soto‐Ortolaza AI , Rayaprolu S , et al. Investigating the role of FUS exonic variants in essential tremor. Parkinsonism Relat Disord. 2013;19:755‐757. 23601511 10.1016/j.parkreldis.2013.03.005 PMC3691340

[25] Ortega‐Cubero S , Lorenzo‐Betancor O , Lorenzo E , et al. Fused in Sarcoma (FUS) gene mutations are not a frequent cause of essential tremor in Europeans. Neurobiol Aging. 2013;34:2441‐2449. 10.1016/j.neurobiolaging.2013.04.024 23731953

[26] Parmalee N , Mirzozoda K , Kisselev S , et al. Genetic analysis of the FUS/TLS gene in essential tremor. Eur J Neurol. 2013;20:534‐539. 23114103 10.1111/ene.12023 PMC4862621

[27] Vande WL , Lamkanfi M , Vandenabeele P . The mitochondrial serine protease HtrA2/Omi: an overview. Cell Death Differ. 2008;15:453‐460. 18174901 10.1038/sj.cdd.4402291

[28] Lin C‐H , Chen M‐L , Chen GS , Tai C‐H , Wu R‐M . Novel variant Pro143Ala in HTRA2 contributes to Parkinson's disease by inducing hyperphosphorylation of HTRA2 protein in mitochondria. Hum Genet. 2011;130:817‐827. 21701785 10.1007/s00439-011-1041-6 PMC3214265

[29] Wang C‐Y , Xu Q , Weng L , et al. Genetic variations of Omi/HTRA2 in Chinese patients with Parkinson's disease. Brain Res. 2011;1385:293‐297. 21338583 10.1016/j.brainres.2011.02.037

[30] Tzoulis C , Zayats T , Knappskog PM , et al. HTRA2 p. G399S in Parkinson disease, essential tremor, and tremulous cervical dystonia. Proc Natl Acad Sci U S A. 2015;112(18):E2268. 25825781 10.1073/pnas.1503105112 PMC4426452

[31] He YC , Huang P , Li QQ , et al. Mutation analysis of HTRA2 gene in chinese familial essential tremor and familial Parkinson's Disease. Parkinsons Dis. 2017;2017:3217474. 28243480 10.1155/2017/3217474 PMC5294371

[32] Suzuki N , Fukushi M , Kosaki K , et al. Teneurin‐4 is a novel regulator of oligodendrocyte differentiation and myelination of small‐diameter axons in the CNS. J Neurosci. 2012;32:11586‐11599. 22915103 10.1523/JNEUROSCI.2045-11.2012 PMC3442259

[33] Houle G , Schmouth J‐F , Leblond CS , et al. Teneurin transmembrane protein 4 is not a cause for essential tremor in a Canadian population. Mov Disord. 2017;32:292‐295. 28158909 10.1002/mds.26753

