# Supplementary material for: Genetic testing of FUS, HTRA2, and TENM4 genes in Chinese patients with essential tremor
Source: CNS Neurosci Ther. 2020 Mar 20;26(8):837–41. doi: 10.1111/cns.13305 (PMC7366735; doi:10.1111/cns.13305)
Supplement: Supplementary file 1 — Table S1‐S3 [file CNS-26-837-s001.doc]

Supplementary table 1

The p-value of all the allele frequency between ET cases and controls for each SNP

| SNP | *p*_1_ | *p*_1’_ | *P*_2_ | *P*_2’_ | *p* | *p*’ |
| --- | --- | --- | --- | --- | --- | --- |
| rs741810 | 0.460 | 0.460 | 0.189 | 0.473 | 0.634 | 0.634 |
| rs1052352 | **0.006** | **0.03** | **0.037** | 0.185 | **0.001** | **0.005** |
| rs11237621 | 0.185 | 0.231 | 0.321 | 0.535 | 0.097 | 0.243 |
| rs689369 | 0.072 | 0.180 | 0.805 | 0.826 | 0.157 | 0.262 |
| rs2277277 | 0.160 | 0.231 | 0.826 | 0.826 | 0.387 | 0.484 |

Note: The positive locus identified in this study was marked in bold font.

Abbreviations: *p*_1_, the p-value of the allele frequency between ET cases with family history and controls; *P*_2_ the p-value of the allele frequency between sporadic ET cases and controls; *p*, the p-value of the allele frequency between all the ET cases and controls; *p*_1’_, *P*_2’_and *p*’, the value was corrected by the FDR test

Supplementary table 2

Power calculation of five loci in FET patients and controls (dominant & recessive models)

| SNP | Model | MAF | Control | FET | CON per case | OR | Power |
| --- | --- | --- | --- | --- | --- | --- | --- |
| rs741810 | Dominant | 0.148 | 132 | 118 | 1.119 | 0.820 | 0.168 |
|  | Recessive |  |  |  |  | 0.556 | 0.137 |
| rs1052352 | Dominant | 0.180 |  |  |  | 1.937 | **0.960** |
|  | Recessive |  |  |  |  | 5.796 | **0.999** |
| rs11237621 | Dominant | 0.528 |  |  |  | 1.291 | 0.217 |
|  | Recessive |  |  |  |  | 1.423 | 0.469 |
| rs689369 | Dominant | 0.140 |  |  |  | 1.697 | 0.803 |
|  | Recessive |  |  |  |  | 1.696 | 0.160 |
| rs2277277 | Dominant | 0.150 |  |  |  | 1.671 | 0.791 |
|  | Recessive |  |  |  |  | 0.368 | 0.266 |

Note: The positive locus identified in this study was marked in bold font.

Abbreviations: CON, controls; MAF, minor allele frequency; OR, odds ratio; FET, essential tremor with family history; SNP, single nucleotide polymorphism.

Supplementary table 3

Power calculation of five loci in SET patients and controls (dominant & recessive models)

| SNP | Model | MAF | Control | SET | CON per case | OR | Power |
| --- | --- | --- | --- | --- | --- | --- | --- |
| rs741810 | Dominant | 0.196 | 140 | 120 | 1.167 | 1.463 | 0.595 |
|  | Recessive |  |  |  |  | 1.169 | 0.0657 |
| rs1052352 | Dominant | 0.160 |  |  |  | 1.675 | **0.828** |
|  | Recessive |  |  |  |  | 4.793 | **0.988** |
| rs11237621 | Dominant | 0.456 |  |  |  | 1.170 | 0.133 |
|  | Recessive |  |  |  |  | 1.333 | 0.301 |
| rs689369 | Dominant | 0.150 |  |  |  | 1.024 | 0.052 |
|  | Recessive |  |  |  |  | 1.769 | 0.210 |
| rs2277277 | Dominant | 0.133 |  |  |  | 1.039 | 0.054 |
|  | Recessive |  |  |  |  | 0.286 | 0.298 |

Note: The positive locus identified in this study was marked in bold font.

Abbreviations: CON, controls; MAF, minor allele frequency; OR, odds ratio; SET, sporadic essential tremor; SNP, single nucleotide polymorphism.
